# Four New Pentasaccharide Resin Glycosides from *Ipomoea cairica* with Strong α-Glucosidase Inhibitory Activity

**DOI:** 10.3390/molecules20046601

**Published:** 2015-04-14

**Authors:** Jie-Tao Pan, Bang-Wei Yu, Yong-Qin Yin, Jie-Hong Li, Li Wang, Li-Bing Guo, Zhi-Bin Shen

**Affiliations:** School of Traditional Chinese Medicinal Chemistry, Guangdong Pharmaceutical University, Guangzhou 510006, China; E-Mails: panjietao123@126.com (J.-T.P.); bondbeth@126.com (B.-W.Y.); lijiehongivy@163.com (J.-H.L.); wangli182646@163.com (L.W.); guolibing512@126.com (L.-B.G.); szb8113@126.com (Z.-B.S.)

**Keywords:** *Ipomoea cairica*, resin glycoside, simonic acid A, α-glucosidase

## Abstract

Six pentasaccharide resin glycosides from *Ipomoea cairica*, including four new acylated pentasaccharide resin glycosides, namely cairicoside I–IV (**1**–**4**) and the two known compounds cairicoside A (**5**) and cairicoside C (**6**), were isolated from the aerial parts of *Ipomoea cairica*. Their structures were established by a combination of spectroscopic, including two dimensional (2D) NMR and chemical methods. The core of the six compounds was simonic acid A, and they were esterfied the same sites, just differing in the substituent groups. The lactonization site of the aglycone was bonded to the second saccharide moiety at C-2 in **1**–**4**, and at C-3 in **5**–**6**. Compounds **1** and **5**, **4** and **6** were two pairs of isomers. The absolute configuration of the aglycone in **1**–**6** which was (11*S*)-hydroxyhexadecanoic acid (jalapinolic acid) was established by Mosher’s method. Compounds **1**–**4** have been evaluated for inhibitory activity against α-glucosidase, which all showed inhibitory activities.

## 1. Introduction

*Ipomoea cairica* (L.) Sweet (Convolvulaceae) is widely distributed in the tropical and subtropical regions and is an invasive species in Southern China. *I. cairica* grows quickly and can then climb up the nearby trees and thus block the light, killing these trees, and damaging small ecological environments [1,2,3]. *I. cairica* was used in folk medicine to treat sores and so on [4]. Many different compounds have been found in the plant, including resin glycosdes, lignans, benzenoids, coumarins, flavonoids, steroids, and fatty acids [5,6]. There were five reported resin glycosides from *I. cairica*, in which the lactone was attached at C-3 of Rha. In this paper six compounds were found, including two pairs of isomers, which were **1** and **5**, and **4** and **6**. The lactone attachment sites in four new resin glycosides **1**–**4** were assigned to C-2 of Rha and the lactone attachment sites of the two known resin glycosides **5**–**6** were assigned to C-3 of Rha. Some plants from the Convolvulaceae family showed strengthened inhibition against α-glucosidase or anti-diabetes activity [7,8], so this paper will elaborate on the structures and α-glucosidase inhibitory activities of compounds **1**–**4**.

## 2. Results and Discussion

Cairicoside I (1) obtained as a white, amorphous powder, which gave a quasi-molecular ion at *m/z* 1321.7212 [M+Na]^+^ in HR-TOF-MS, which suggested the molecular formula C_65_H_102_O_26_ (calcd. for C_65_H_102_O_26_Na, 1321.6557). Its IR spectrum gave a hydroxyl group absorption band at 3442 cm^−1^, carbonyl group at 1724 cm^−1^, and aromatic group at 1641 cm^−1^. Alkaline hydrolysis of **1** afforded a glycosidic acid (compound **7**) and organic acids. The organic acids were identified as 2-methylbutyric, and Cna (*trans*-cinnamic acids), with a ratio of 2:1 by GC-MS. Mba^a^ (2-Methylbutyric acid) was found to have the *S*-configuration by comparison of its optical rotation value with that of an authentic sample [3]. Acid hydrolysis the glycosidic acid afforded Rha (l-rhamnose) and Glu (d-glucose), with a ratio of 4:1. The monosaccharides was derivatized and identified as Rha and Glu by GC-MS by comparison with authentic samples. Taken together the hydrolysis information and NMR data identified the glycosidic acid as simonic acid A (**7**).

**Figure 1:**
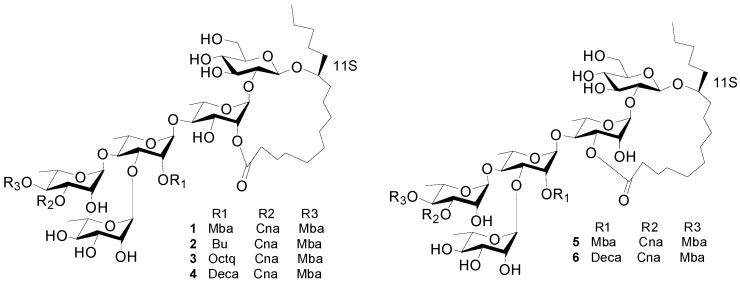
The structures of compounds **1**–**6**.

The ^1^H-NMR spectrum of **1** (Figure 1) exhibited two *trans*-coupled olefinic protons at δ_H_ 6.53 (1H, d) and 7.81 (1H, d), a multiplet due to five protons at δ_H_ 7.27–7.45 (m) suggesting the presence of a Cna. The protons at δ_H_ 0.82 (1H, t), 1.13 (1H, d), and 2.38 (m) were assignable to another Mba group. The ^13^C-NMR spectrum data of **1** (Table 1) exhibited four ester carbonyl carbons at δ_C_ 175.7, 175.2, 173.7, and 166.1 and five anomeric carbon signals at δ_C_ 104.6, 104.3, 103.6, 99.2 and 98.3. The anomeric protons were assigned to the peaks at 5.66 (1H, br s), 4.91 (1H, d), 5.94 (1H, br s), 6.08 (1H, br s) and 5.58 (1H, br s), respectively, by the HSQC data. All protons in each saccharide system and carbon signals were assigned by ^1^H-, ^13^C-NMR, TOCSY, HMBC and HSQC experiments, leading to the identification of one glucopyranosyl unit and four rhamnopyranosyl units as the monosaccharides present in **1**. The anomeric configuration for the sugar moieties were defined as β for the glucopyranosyl moieties from their coupling constants of 7.2 Hz, and α for the rhamnopyranosyl from the C-5 chemical shift [9].

**Table 1:** NMR Data for Compounds **1**–**4** in pyridine-*d*_5_.

Position ^b^	1 ^a^			2 ^a^		3 ^a^		4 ^a^	
	^13^C	^1^H	^13^C	^1^H	^13^C	^1^H	^13^C	^1^H
Glc-1	104.3	4.91 d (7.2)	104.3	4.97 d (7.2)	102.7	4.95 d (7.2)	104.3	4.91 d (7.2)
2	81.8	3.87 *	81.8	3.95 *	80.3	3.92 *	81.8	3.89 *
3	76.3	4.17 dd (9.0, 9.0)	76.1	4.24 dd (9.0, 9.0)	74.8	4.21 dd (9.0, 9.0)	76.3	4.21 dd (9.0, 9.0)
4	71.7	4.12 dd (9.0, 9.0)	71.7	4.12 dd (9.0, 9.0)	70.2	4.15 dd (9.0, 9.0)	71.7	4.12 dd (9.0, 9.0)
5	77.8	3.83 *	77.8	4.18 *	76.3	3.90 *	77.8	3.84 *
6a	62.6	4.44–4.46 *	62.6	4.52 *	61.1	4.48 *	62.6	4.48 *
6b		4.29 *		4.34 *		4.30 *		4.30 *
Rha-1	98.3	5.58 br s	98.5	5.64 br s	97.0	5.61 br s	98.6	5.58 br s
2	71.2	6.04 br s	71.2	6.16 br s	69.7	6.15 br s	71.2	6.15 br s
3	69.6	5.05 dd (9.6, 3.0)	69.6	5.12 dd (9.6, 2.0)	67.9	5.10 dd (9.6, 3.0)	69.5	5.08 dd (9.6, 2.0)
4	79.8	4.18 dd (9.6, 9.6)	79.4	4.24 dd (9.6, 9.6)	77.8	4.23 dd (9.6, 9.6)	79.3	4.20 dd (9.6, 9.6)
5	68.2	4.24 *	68.1	4.27 *	66.6	4.28 *	68.1	4.30 *
6	19.2	1.56 d (6.0)	18.6	1.60 d (6.0)	18.6	1.59 d (6.0)	18.6	1.59 d (6.0)
Rha'-1	99.2	6.08 br s	99.3	6.12 br s	97.6	6.08 br s	99.0	6.05 br s
2	72.8	5.98 br s	72.9	6.06 br s	71.3	6.05 br s	73.0	6.02 br s
3	79.4	4.56 dd (9.0, 2.4)	79.9	4.56 dd (9.0, 2.4)	78.3	4.66 dd (10.0, 2.4)	79.8	4.64 dd (9.0, 2.4)
4	80.2	4.29 dd (9.0, 9.0)	79.4	4.20 dd (9.0, 9.0)	78.8	4.20 dd (10.0, 10.0)	80.2	4.20 dd (9.0, 9.0)
5	68.1	4.34 dd (9.0, 6.0)	68.1	4.54 *	66.6	4.53 *	68.0	4.50 *
6	18.4	1.64 d (6.0)	18.3	1.67 d (5.5)	16.1	1.64 d (5.4)	17.6	1.64 d (5.4)
Rha"-1	103.6	5.94 br s	103.1	5.94 br s	101.8	5.99 br s	103.4	5.96 br s
2	72.4	4.78 br s	72.4	4.84 br s	70.9	4.81 br s	72.4	4.77 br s
3	73.2	5.91 dd (3.0, 10.0)	73.1	5.96 dd (3.0, 10.0)	71.4	5.93 dd (2.9, 10.2)	73.1	5.90 dd (3.0, 10.0)
4	73.5	6.06 t (10.0)	73.5	6.11 t (10.0)	71.8	6.10 dd (10.2, 10.2)	73.4	6.05 dd (10.0, 10.0)
5	68.4	4.49 dd (10.0, 6.5)	68.4	4.30 *	66.9	4.30 *	68.4	4.21 *
6	17.6	1.36 d (6.5)	19.2	1.46 d (6.0)	17.7	1.43 d (6.0)	19.2	1.42 d (5.5)
Rha"'-1	104.6	5.66 br s	104.3	5.69 br s	102.7	5.65 br s	104.3	5.62 br s
2	69.8	4.97 br s	69.8	4.97 br s	68.3	4.98 br s	69.8	4.94 br s
3	72.3	4.39 *	72.3	4.37 *	70.9	4.38 *	72.3	4.35 *
4	73.2	4.23 *	73.4	4.24 *	71.8	4.23 *	73.4	4.21 *
5	70.4	4.41 *	70.6	4.45 *	69.1	4.43 *	70.6	4.40 *
6	18.6	1.58 d (6.0)	18.5	1.58 d (6.0)	16.9	1.61 d (6.0)	18.4	1.59 d (6.0)
Ag-1	173.7		173.5		171.6		173.1	
2	33.0	2.40 m, 2.24 m	34.2	2.30 m, 2.24 m	32.6	2.40 m, 2.29 m	33.0	2.40 m 2.26 m
Ag-1	173.7		173.5		171.6		173.1	
2	33.0	2.40 m, 2.24 m	34.2	2.30 m, 2.24 m	32.6	2.40 m, 2.29 m	33.0	2.40 m 2.26 m
11	82.5	3.89 *	82.6	3.96 *	81.1	3.90 *	82.6	3.90 *
16	14.0	0.84 t (7.0)	14.0	0.85 t (7.0)	12.6	0.82 t (7.0)	14.1	0.84 t (7.0)
Cna-1	166.1		166.0		164.8		166.2	
2	118.1	6.53 d (16.0)	118.0	6.60 d (16.0)	116.8	6.57 d (16.0)	118.1	6.54 d (16.0)
3	145.4	7.81 d (16.0)	145.3	7.86 d (16.0)	143.9	7.84 d (16.0)	145.3	7.75 (16.0)
1'	134.3		134.4		133.0		134.4	
2' and 6'	128.4	7.43 m	128.3	7.49 m	126.8	7.46 m	128.3	7.44 m
3' and 5'	129.0	7.34 m	129.1	7.38 m	127.6	7.36 m	129.1	7.34 m
4'	130.7	7.34 m	130.4	7.38 m	129.0	7.36 m	130.5	7.34 m
Mba-1	175.2		175.6		175.7		175.7	
2	41.2	2.38 m	41.3	2.45 m	39.8	2.49 m	41.3	2.47 m
2-CH_3_	16.6	1.06 d (7.0)	16.7	1.17 d (7.0)	15.2	1.13 d (7.0)	16.7	1.13 d (7.0)
3	27.5	1.41 m *	27.5	1.41 m *	27.4	1.38 m *	27.1	1.44 m *
4	11.6	0.81 t (7.0)	11.7	0.79 t (7.0)	10.1	0.79 t (7.0)	11.6	0.79 t (7.0)
Mba'-1	175.7							
2	41.3	2.47 m						
2-CH_3_	16.7	1.13 d (7.0)						
3	27.0	1.22 m *						
4	11.6	0.82 t (7.0)						
Bu-1			172.5					
2			17.6	2.50 tq (7.0, 7.0)				
4			13.4	0.84 t (7.0)				
Oct-1					171.3			
2					32.6	2.30 m		
8					12.5	0.81 t (7.0)		
Deca-1							172.7	
2							34.2	2.32 tq (7.0, 7.0)
10							14.0	0.83 t (7.0)

^a^ Chemical shifts (δ) are in ppm relative to TMS. The spin coupling (*J*) is given in parentheses (Hz). Chemical shifts marked with an asterisk (*) indicate overlapped signals. Spin-coupled patterns are designated as follows: s = singlet, br s = broad singlet, d = doublet, t = triplet, m = multiplet, q = quartet. All assignments are based on ^1^H-^1^H TOCSY experiments. ^b^ Abbreviations:Glc = glucose; Rha = rhamnose; Ag = 11-hydroxyhexadecanoyl; Mba = 2*S*-methylbutanoyl; CA = *trans*-cinnamoyl; Deca = *n*-decanoyl; Octa = *n*-octanoyl; Bu = butytyl.

**Figure 2:**
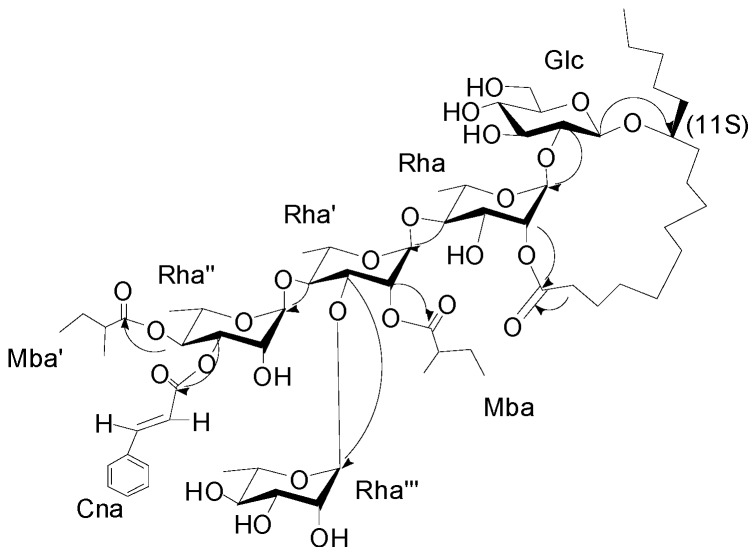
Key HMBC correlations from H to C for cairicoside I (**1**).

The interglycosidic connectivities were determined from the following HMBC (Figure 2) correlations from Rha-H-1 (δ_H_ 5.58) to Glu-C-2 (δ_C_ 81.8), Rha'-H-1 (6.08) to Rha-C-4 (79.8), Rha''-H-1 (δ_H_ 5.94) to Rha'-C-4 (80.2), Rha'''-H-1 (δ_H_ 5.66) to Rha'-C-3 (79.4). Esterification positions were also determined by HMBC data, between Rha''-H-4 (δ_H_ 6.06) to Mba-C-1(δ_C_ 175.7); Rha''-H-3 of rhamnose'' (δ_H_ 5.91) to Cna-C-1 (δ_C_ 166.1); Rha'-H-2 (δ_H_ 5.98) to Mba-C-1 (δ_C_ 175.2); and Rha-H-2 (δ_H_ 6.04) and aglycone-C-1 (δ_C_ 173.7), respectively. The position of the jalapinolic acid unit was finally determined by HMBC to be between aglycone-H-11 (δ_H_ 3.89) to Glu-C-1 (δ_C_ 104.3), which correlations established the structure of **1** as (*S*)-jalapinolic acid 11-*O*-α-l-rhamnopyranosyl-(1→3)-*O*-[3-O-trans-cinnamoyl-4-O-(*S*)-2-methyl-butyryl-α-l-rhamnopyranosyl-(1→4)]-*O*-[2-O-(*S*)-2-methylbutyryl]-α-l-rhamnopyranosyl-(1→4)-*O*-α-l-rhamnopyranosyl-(1→2)-*O*-β-d-glucopyranoside, intramolecular 1,2''-ester (Figure 1). This new compound was named cairicoside I.

Cairicosides II–IV (compounds **2**–**4**) afforded white, amorphous powders, and gave quasi-molecular ions at *m/z* 1307.6437 [M+Na]^+^, 1363.7032 [M+Na]^+^ and 1391.7395 [M+Na]^+^ in HR-TOF-MS, which suggested the molecular formulas C_64_H_100_O_26_ (calcd. for C_64_H_100_O_26_Na: 1391.7340), C_65_H_101_O_26_ (calcd. for C_68_H_108_O_26_Na: 1363.7027) and C_70_H_112_O_26_ (calcd. for C_70_H_112_O_26_Na: 1391.7340). The IR spectra gave absorption bands of hydroxyl groups at 3418, 3443 and 3444 cm^−1^, carbonyl groups at 1721, 1740 and 1740 cm^−1^, and aromatic groups at 1641, 1638 and 1638 cm^−1^. Independent alkaline hydrolysis of **2**–**4** afforded a mixture of organic acids and a glycosidic acid, respectively. 2*S*-Methylbutanooic acid and a *trans*-cinnamoic acid were found in **2**–**4** by GC-MS experiments. Additionally, a butyl acid in **2**, a *n*-octanoyl acid in **3** and a *n*-decanoyl acid were found in **4** by GC-MS expriments. The glycosidic acid obtained also proved to be simonic acid A (**7**) from the NMR and MS data. The ^1^H-NMR spectrum of **2** exhibited two *trans*-coupled olefinic protons at δ_H_ 6.60 (1H, d, *J* = 16.0 Hz, Cna-2) and 7.86 (1H, d, *J* = 16.0 Hz Cna-3), a multiplet due to five protons at δ_H_ 7.38–7.49 (m, Cna-2-6), suggesting the presence of a *trans*-cinnamoyl moiety. The same *trans*-cinnamoyl moiety appeared in **3**–**4**, two *trans*-coupled olefinic protons at δ_H_ 6.57 (1H, d, *J* = 16.0 Hz, Cna-2) and 7.84 (1H, d, *J* = 16.0 Hz Cna-3), a multiplet due to five protons at δ_H_ 7.36–7.46 (m, Cna-2-6) in **3**, and two *trans*-coupled olefinic protons at δ_H_ 6.54 (1H, d, *J* = 16.0Hz, Cna-2 ) and 7.75 (1H, d, *J* = 16.0 Hz, Cna-3), a multiplet due to five protons at δ_H_ 7.34–7.44 (m, Cna-2-6) in **4**. The protons at δ_H_ 0.79 (1H, t, *J* = 7.0 Hz, Mba-4), 1.17 (1H, d, *J* = 7.0 Hz, Mba-2-Me), and 2.45 (m, Mba-2) were assignable to a 2-methylbutanoyl (Mba) group in **2** and the protons at δ_H_ 0.79 (1H, t, *J* = 7.0 Hz, Mba-4), 1.13 (1H, d, *J* = 7.0 Hz, Mba-2-Me), and 2.49 (m, Mba-2) suggested an Mba group in **3**, and δ_H_ 0.79 (1H, t, *J* = 7.0 Hz, Mba-4), 1.13 (1H, d, *J* = 7.0 Hz, Mba-2- CH_3_), and 2.47 (m, Mba-2) were also ive of an Mba group in **4**. Alkaline hydrolysis **2**–**4** gave an organic acid mixture and one glycosidic acid, which were identified as 2-methylbutyric and *trans*-cinnamic acids in **2**–**4**, butyl acid in **2**, *n*-octanoyl acid in **3** and *n*-decanoyl acid in **4** by GC-MS expriments. The ^13^C-NMR spectrum of **2**–**4** were highly similar from δ_C_ 180 to δ_C_ 40 with **1**, so the three compounds should have very similar connection positions, just differing in the substituents. The key HMBC correlations confirmed the esterification positions of the acyl residues in the oligosaccharide core, thus a *2S-*methylbutanoyl group and a *trans*-cinnamoyl group were located at C-4 of Rha" in **2**–**4**. A butyl, *n*-octanoyl and *n*-decanoyl group were located at C-2 of Rha', in **2**, **3** and **4**, respectively. The lactonization position of the aglycone was C-2 of Rha for **2**–**4**. Accordingly, the structures of **2**–**4** were as shown in Figure 1.

Cairicoside A (**5**) and cairicoside C (**6**) appeared as white, amorphous powders, and gave quasi-molecular ions at *m/z* 1333 [M+Cl]^−^, and 1403 [M+Cl]^−^ in ESI-MS, which suggested molecular weights of 1298 and 1368, so these two compounds were isomeric with **1** and **4**, respectively. Alkaline hydrolysis of **5** afforded the same substituent groups as **1**, and alkaline hydrolysis of **6** afforded the same substituent groups as **4**. The groups’ esterfication sites were suggested by HMBC data, and just the lactone sites were different between **1** and **4**, which were bonded at C-3 of Rha. The structures of **5**–**6** are shown in Figure 1.

Some plants from the Convolvulaceae family have been reported to exert anti-diabetes activities or potent α-glucosidase inhibitory activities, so compounds **1**–**4** have been evaluated for inhibitory activities against α-glucosidase. As shown in Table 2, new compounds **1**–**4** exhibited more potent α-glucosidase inhibitory activities compared to acarbose, a widely used clinically useful drug, used as a positive control (IC_50_ = 385.0 ± 9.3 μM). To our knowledge, these are the first examples of resin glycosides with α-glucosidase inhibitory activities. The IC_50_ values of the four compounds are almost the same, probably due to the similarity of their structures, and every compound with strong inhibitory activity has four α-anomeric monosaccharides in the structure, which is the same relative configuration as α-glucosidase. 

**Table 2:** α-Glucosidase inhibition of compounds **1**–**4**.

Compound	α-Glucosidase Inhibition Contstant ^a^
	(IC_50_) [μM]
1	21.4 ± 2.9
2	26.2 ± 4.6
3	30.4 ± 3.9
4	28.9 ± 1.4
Acarbose ^b^	385.0 ± 9.3

^a^ IC_50_ is defined as the concentration that resulted in a 50% α-glucosidase inhibition and the results are means ± standard deviation of three independent replicates; ^b^ Positive control substance.

## 3. Experimental Section

### 3.1. General Information

^1^H- and ^13^C-NMR spectra were recorded on an INOVA 600 spectrometer using tetramethylsilane (TMS) as internal standard. The chemical shifts were given in δ (ppm) and coupling constants in Hz. The ESIMS experiments were performed on an Agilent 1100 Series LC/MSD ion-trap mass spectrometer. HR-TOF-MS experiments were performed on AB SCIEX Triple TOF 5600 plus MS spectrometer. UV were measured on a Shimadzu UV-2550 spectrophotometer and IR spectra were measured on a Shimadzu FTIR spectrophotometer. GC-MS experiments were performed on a TRACE GC ULTRA DSQ II instrument. Optical rotations were measured with an Anton Paar-MCP600 polarimeter in MeOH solution. The centrifugation was performed with a Beckman Allegra X-12). Adsorbents for column chromatography were silica gel (200–300 μm, Qingdao Marine Chemical Co., Ltd., Qingdao China), Sephadex LH-20 (75–150 μm, Pharmacia, Uppsala, Sweden), ODS (40–63 μm, FuJi, Tokyo, Japan). Preparative high-performance liquid chromatography (HPLC) was performed using a Shimadzu LC-6ADseries instrument equipped with a UV detector at 280 nm and Shim-Park RP-C_18_ column (20 × 200 mm i.d.). TLC (Thin-layer chromatography) was performed on pre-coated silica gel GF_254_ plates (Qingdao Marine Chemical Co., Ltd.) and detected by spraying with 10% H_2_SO_4_–EtOH. α-Glucosidase was monitored continuously with an auto multi-functional microplate reader ELX800 (BioTek Instruments, Inc., Winooski, VT, USA).

### 3.2. Plant Material 

The aerial parts of *I*. *cairica* were collected at Guangzhou City, Guangdong Province, China, in August 2012, and identified by Prof. Ji-Zhu Liu. A voucher specimen (No. 2012-8) was deposited at Department of Traditional Chinese Medicinal Chemistry, Guangdong Pharmaceutical University. 

### 3.3. Extraction and Isolation

The aerial parts of *I*. *cairica* (5 kg) were extracted two times with 95% EtOH (20 L) under reflux for 2 h and then evaporated *in vacuo*. The extract was divided into CHCl_3_ and H_2_O-soluble fractions. The CHCl_3_ extract (107 g) was subjected to column chromatography on silica gel (200–300 mesh, 2 kg, 10 × 80 cm) and eluted with CH_2_Cl_2_–MeOH (v/v, 100:0, 20:1, 5:1) to give three fractions (Fr.1-3). Fr.2 (13 g) was purified by Sephadex LH-20 (75–150 μm, 120 g, 1.5 × 160 cm) column chromatography with MeOH as eluent, fractions of 10–19 (3.8 g) were put together as Fr. A1 and fractions of 3–9 were combined as Fr. A2. Fr. A1 was purified by a reverse-phase HPLC system (10 mL/min, 280 nm), eluted with MeOH/H_2_O (v/v, 95:5) to furnish **1** (52 mg), **2** (9.3 mg), **5** (34 mg) and **6** (17 mg). Fr. A2 was purified by the same system, eluted with MeOH/H_2_O (v/v, 98:2) to yield **3** (32 mg), and **4** (40 mg).

### 3.4. Product Identification

*Cairicoside I* (**1**), white amorphous powder, ${\left[\alpha \right]}_{D}^{25}$ + 53.8° (*c* 0.3, MeOH). UV (MeOH) λ_max_: (log ε) 278 (0.657), 216 (0.402) nm; IR (KBr) ν_max_: 3442, 2931, 2859, 2334, 1724, 1641, 1134, 1058 cm^−1^; ^1^H- and ^13^C-NMR (C_5_D_5_N) data see Table 1; HR-TOF-MS *m/z* 1321.6542 [M+Na]^+^ (calcd for C_65_H_102_O_26_Na: 1321.6557).

*Cairicoside II* (**2**), white amorphous powder, ${\left[\alpha \right]}_{D}^{25}$ + 30.5° (*c* 0.1, MeOH). UV (MeOH) λ_max_: (log ε) 280 (0.26), 226 (0.54) nm; IR (KBr) ν_max_: 3418, 2938, 1721, 1641, 1082 cm^−1^; ^1^H- and ^13^C-NMR (C_5_D_5_N) data see Table 1; HR-TOF-MS *m/z* 1307.6437 [M+Na]^+^ (calcd for C_64_H_100_O_26_Na: 1307.6401).

*Cairicoside III* (**3**), white amorphous powder, ${\left[\alpha \right]}_{D}^{25}$ + 8.7° (*c* 0.2, MeOH). UV (MeOH) λ_max_: (log ε) 278 (0.695), 220 (0.485) nm; IR (KBr) ν_max_: 3444, 2931, 2858, 1739, 1638, 1054 cm^−1^; ^1^H- and ^13^C-NMR (C_5_D_5_N) data see Table 1; HR-TOF-MS *m/z* 1363.7032 [M+Na]^+^ (calcd for C_68_H_108_O_26_Na: 1363.7027).

*Cairicoside IV* (**4**), white amorphous powder, ${\left[\alpha \right]}_{D}^{25}$ + 88.3° (*c* 0.10, MeOH); UV (MeOH) λ_max_ 220 (0.34), 278 (0.56) nm; IR (KBr) ν_max_: 3444, 2931, 2858, 1740, 1638, 1054 cm^−1^; ^1^H- and ^13^C-NMR (C_5_D_5_N) data see Table 1; HR-TOF-MS *m/z* 1391.7395 [M+Na]^+^ (calcd for C_70_H_112_O_26_Na:1391.7340).

*Cairicoside A* (**5**), white amorphous powder, ${\left[\alpha \right]}_{D}^{25}$ − 54.0° (*c* 0.10, MeOH); UV (MeOH) λ_max_ 222 (0.33), 278 (0.58) nm; IR (KBr) ν_max_: 3439, 2933, 2863, 1727, 1637, 1053 cm^−1^; ESIMS *m/z* 1333 [M+Cl]^−^. 

*Cairicoside C* (**6**), white amorphous powder, ${\left[\alpha \right]}_{D}^{25}$ − 58.1° (*c* 0.11, MeOH); UV (MeOH) λ_max_ (log ε) 216 (0.4), 278 (0.66) nm; IR (KBr) ν_max_: 3442, 2931, 2859, 1724, 1641, 1058 cm^−1^; ESIMS *m/z* 1403 [M+Cl]^−^.

### 3.5. Alkaline Hydrolysis of **1**–**6**

Compounds **1**–**6** (7 mg each) in 5% KOH (3 mL) were refluxed at 90 °C for 2 h, respectively. The reaction mixture was acidified to pH 4.0 with 2 mol/L HCl and extracted with hexane (3 mL × 2) and *n*-BuOH (3 mL × 2). The organic layer was washed with H_2_O, dried over anhydrous Na_2_SO_4_, then methylated following [10]. The hexane extract, was combined with 0.1 mL 0.5 M CH_3_ONa solution, then shaken for 5 min at room temperature, before adding 5 μL CH_3_COOH and 1 g anhydrous CaCl_2_ powder, heating for 1 h, followed by centrifugation for 2–3 min at 2000–3000 rpm.min^−1^. The supernatant was analyzed by GC-MS on a TRACE GC ULTRA DSQ II intrument under the following conditions: 30 m × 0.25 mm × 0.25 μm, TG-5MS (Thermo) column; He, 0.8 mL/min; 40 °C, 3min; 50–310 °C, ∆10 °C/min, 70 eV. 2-Methylbutyric acid methyl ester (*t*_R_ 4.39 min) *m/z* [M+H]^+^ 117 (5), 101 (23), 88 (96), 57 (100), 41 (55), 29 (45), 27 (19), and *trans*-cinnamic acid methyl ester (*t*_R_ 13.29 min) *m/z* [M]^+^ 162 (40), 131 (100), 103 (66), 77 (32), from **1**–**6** was identified. *n*-Butyric acid methyl ester (*t*_R_ 4.37 min) *m/z* [M]^+^ 101 (33), 88 (100), 57 (70), 41 (35) from **2**. *n*-Octanoic acid methyl ester (*t*_R_ 10.82 min): *m/z* [M]^+^ 158 (4), 127 (18), 87 (45), 74 (100), 43 (22) from **3** was identified. *n*-Decanoic acid methyl ester (*t*_R_ 12.37 min): *m/z* 172 [M]^+^ (4), 155 (5), 143 (30), 129 (5), 87 (59), 74 (100), 55 (18) from **4** and **6** was identified. The 2-methylbutanoic acid was proved to have an *S* configuration by comparing the specific rotation with that of authentic 2*S*-methylbutanoic acid [11].

### 3.6. Acid Hydrolysis and Sugar Analysis

The glycosidic acid (**7**, 4 mg, from alkaline hydrolysis) was methylated with MeOH catalyzed by 1.0 mol/L H_2_SO_4_ to give simonic acid A methyl ester (**8**). Compound **7** was hydrolyzed with 2 mol/L H_2_SO_4_ and extracted with ether to obtain 11-hydroxyhexadecanoic acid methyl ester (**9**) [12]. The aqueous layer of acidic hydrolysis was concentrated under reduced pressure to give a sugar residue. The protocols described in [13] were applied to determine the stereochemistry of sugars, which allowed the identification of the components of the mixture of sugars as l-rhamnose and d-glucose by comparison their derivatives with those of authentic samples. GC-MS was performed on a TRACE GC ULTRA DSQ II intrument under the following conditions: 30 m × 0.25 mm × 0.25 μm, TG-5MS (Thermo)column; He, 0.8 mL/min; 60 °C, 3 min; 60–180 °C, ∆10 °C/min keep 3 min, 180–205 °C, ∆3 °C/min keep 5 min, 205–300 °C, ∆20 °C/min keep 5 min, 70 eV. In the acid hydrolysate of operculinic acid A methyl ester l-rhamnose, and d-glucose were confirmed by comparison of their retention times of their derivatives with those of authentic l-rhamnose (*t*_R_ 30.14 min) and d-glucose (*t*_R_ 31.65 min) derivatives prepared in the same way, respectively.

### 3.7. Preparation of Mosher’s Esters

The procedures for preparation of Mosher’s esters to determination of absolute configuration of 11*S* of the aglycone were same as described previously for resin glycosides from *Ipomoea batatas* [10]. According to the ∆δ of the derivative of **9** with *R*-MPA and *S*-MPA, so compound **9** was assigned to 11*S*.

### 3.8. Enzyme Inhibition Assay

The a-glucosidase inhibition assay was performed according to a slightly modified method of Pierre *et al.* [14]. α-Glucosidase was purchased from Sigma (San Francisco, CA, USA, No. M0035-15). The inhibition was measured spectrophotometrically at pH 6.8 and at 37 °C for 10 min, using 0.01 M *p*-nitrophenyl a-d-glucopyranoside (PNPG) from Sigma (No. M0103) as a substrate and 1 U/mL of enzyme, in 0.067 M KH_2_PO_4_-Na_2_HPO_4_ buffer. Acarbose (Aladdin Company, Shanghai, China, No. l1424006) was used as positive control. The increment in absorption at 410 nm due to the hydrolysis of PNPG by α-glucosidase was monitored continuously with an automatic multi-functional microplate reader.

## 4. Conclusions

The new compounds **1**–**4** were isolated from *Ipomoea cairica* and identified. These new compounds displayed strong α-glucosidase inhibitory activity.

## Supplementary Materials

Supplementary materials can be accessed at: http://www.mdpi.com/1420-3049/20/04/6601/s1.
