# Supplementary material for: Four New Pentasaccharide Resin Glycosides from Ipomoea cairica with Strong α-Glucosidase Inhibitory Activity
Source: Molecules. 2015 Apr 14;20(4):6601–10. doi: 10.3390/molecules20046601 (PMC6272348; doi:10.3390/molecules20046601)
Supplement: Supplementary file 1 [file molecules-20-06601-s001.pdf]

Supplementary Materials

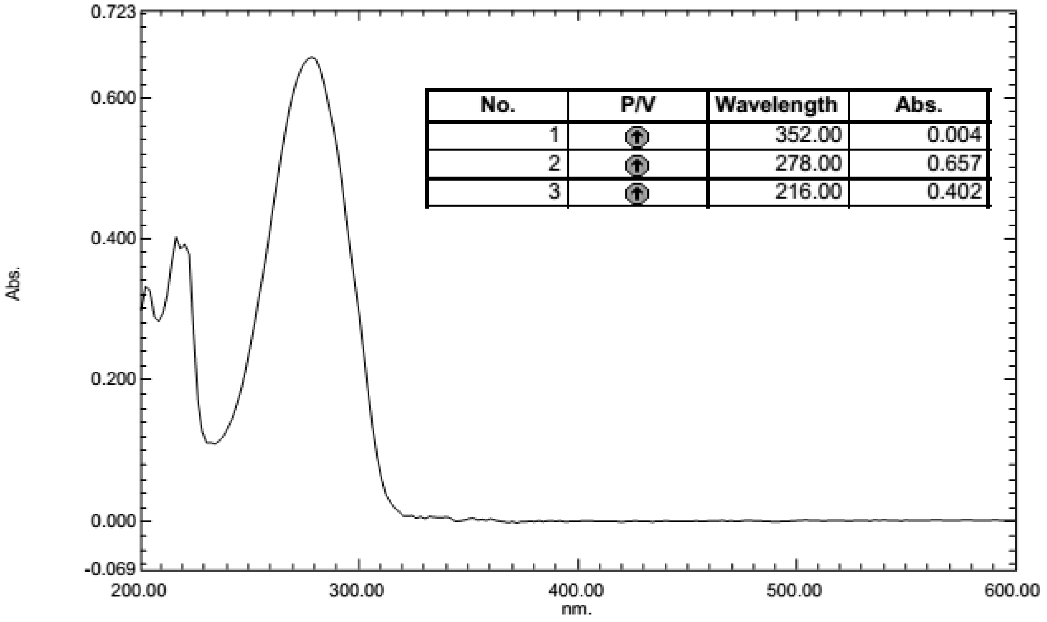

Figure S1. The UV spectrum compounds **1**.

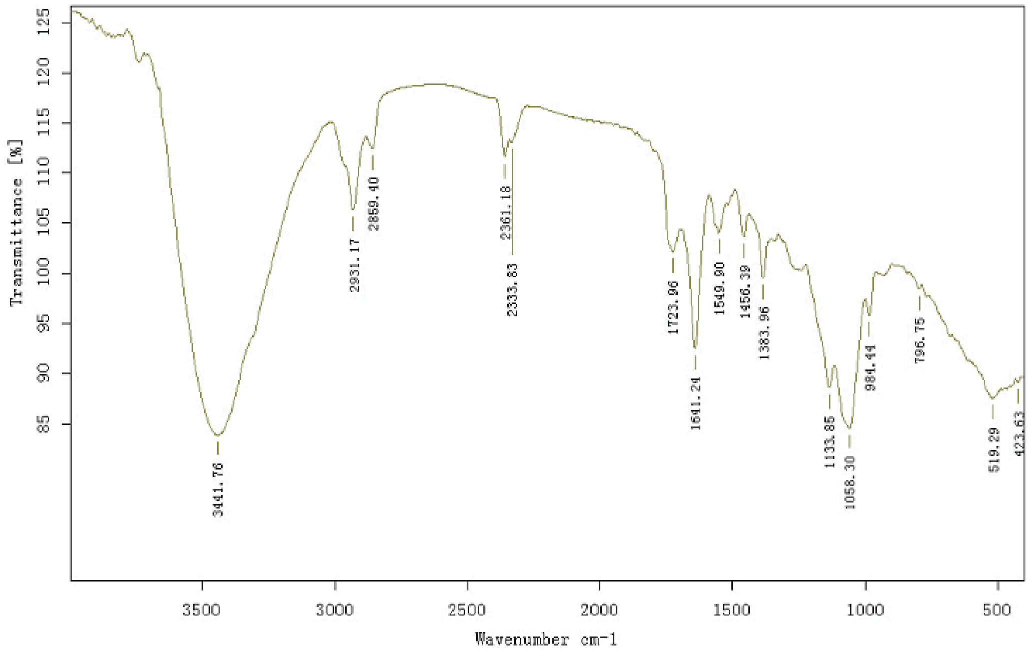

Figure S2. The IR spectrum compounds **1**.

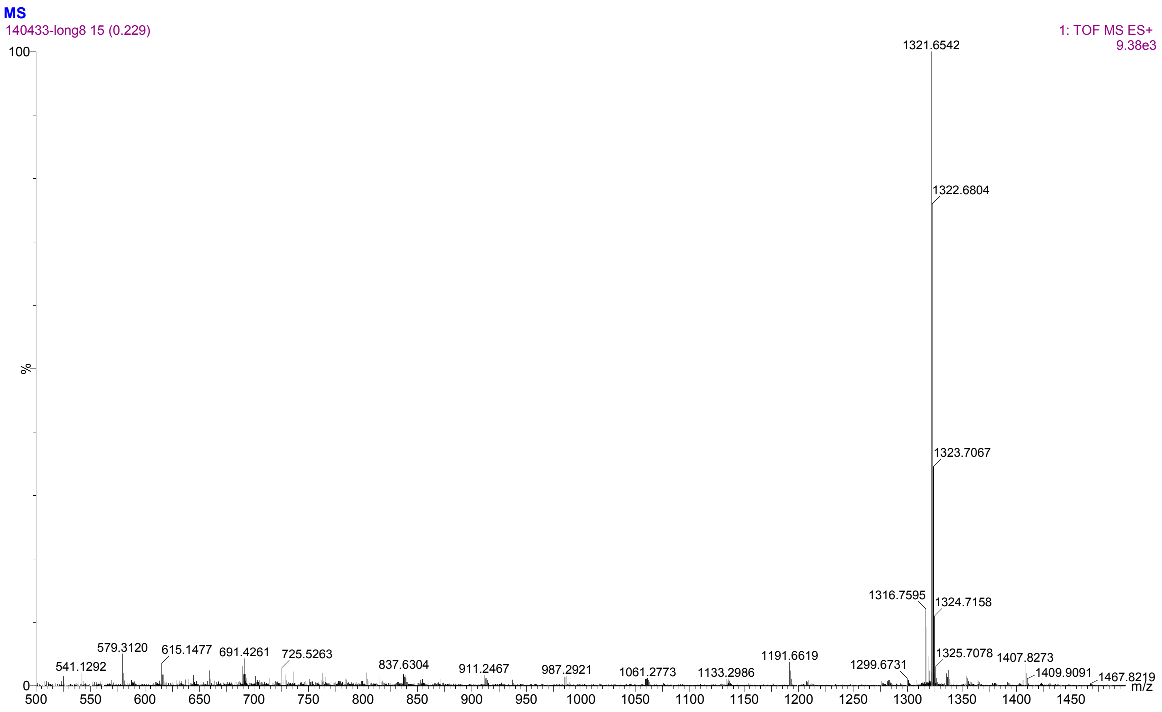

Figure S3. The HR-TOF-MS spectrum compounds **1**.

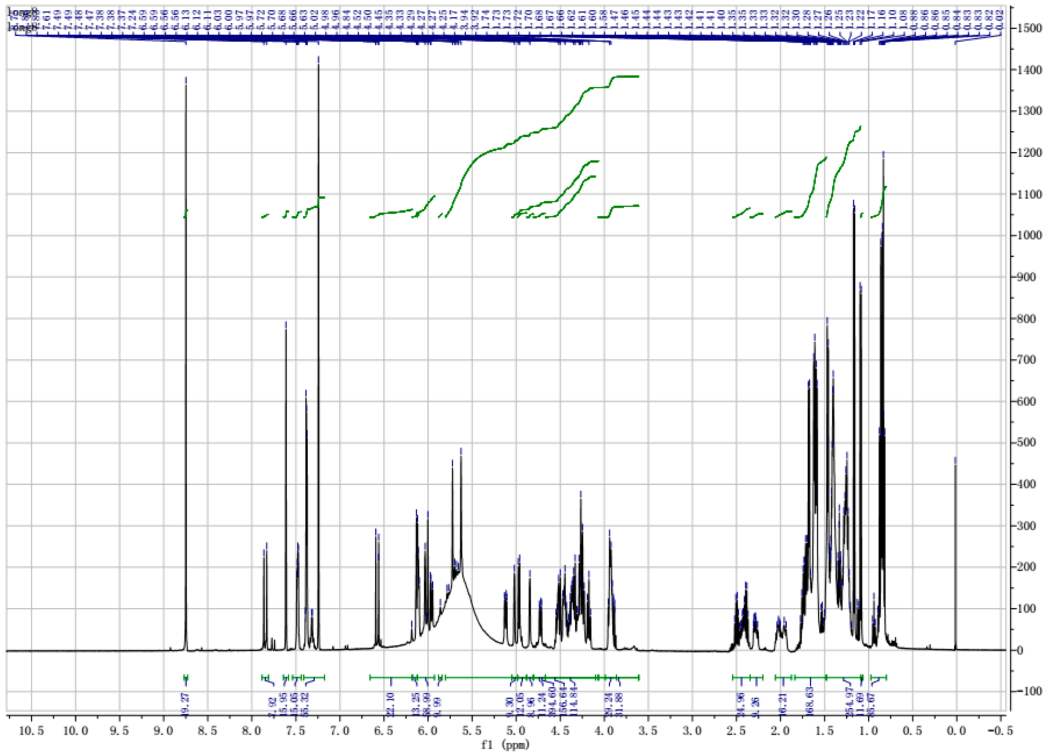

Figure S4. The ^{1}H-NMR spectrum compounds **1**.

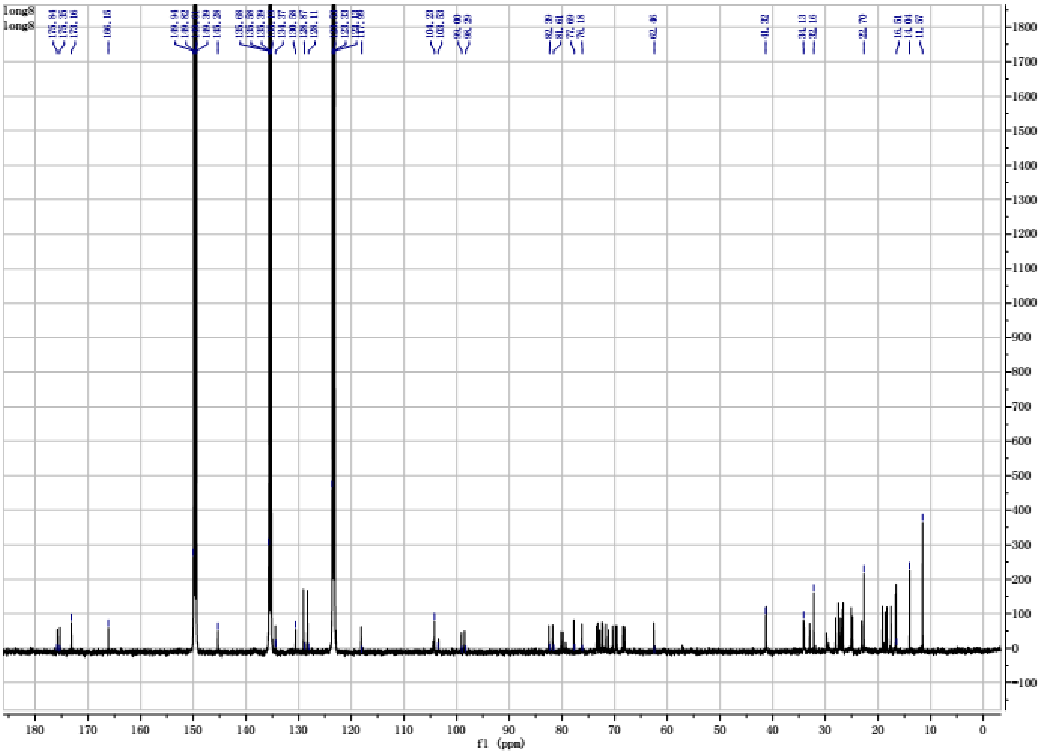

Figure S5. The ^{13}C-NMR spectrum compounds **1**.

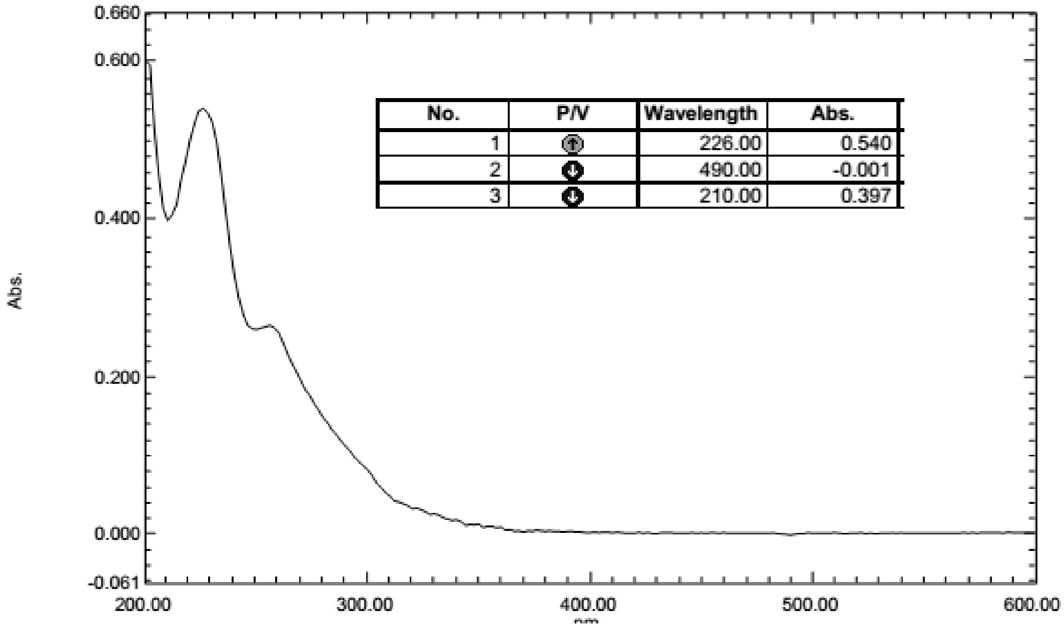

Figure S6. The UV spectrum compounds **2**.

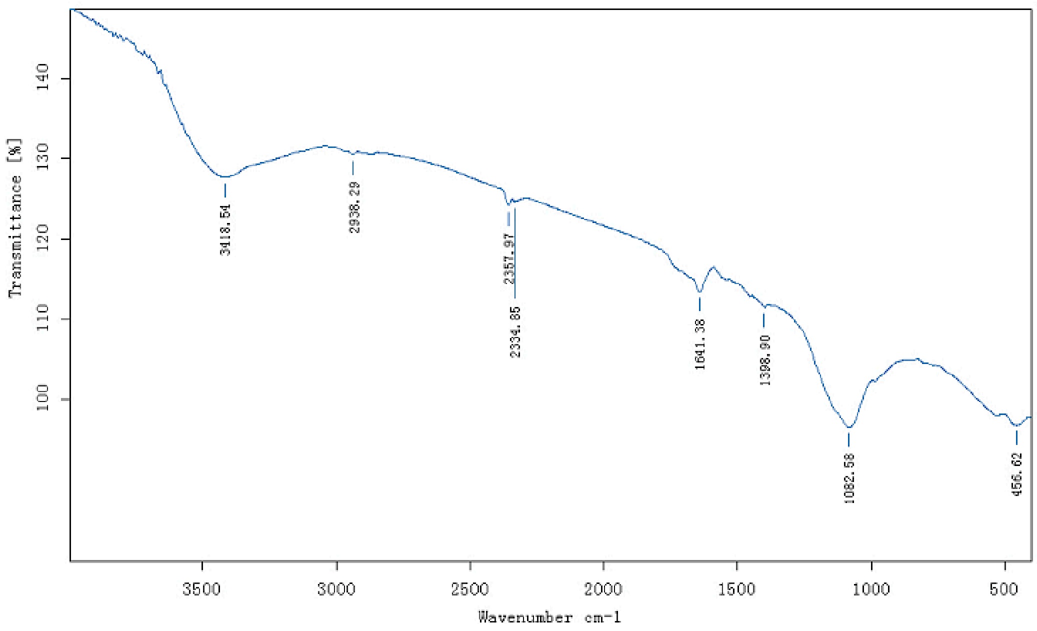

Figure S7. The IR spectrum compounds **2**.

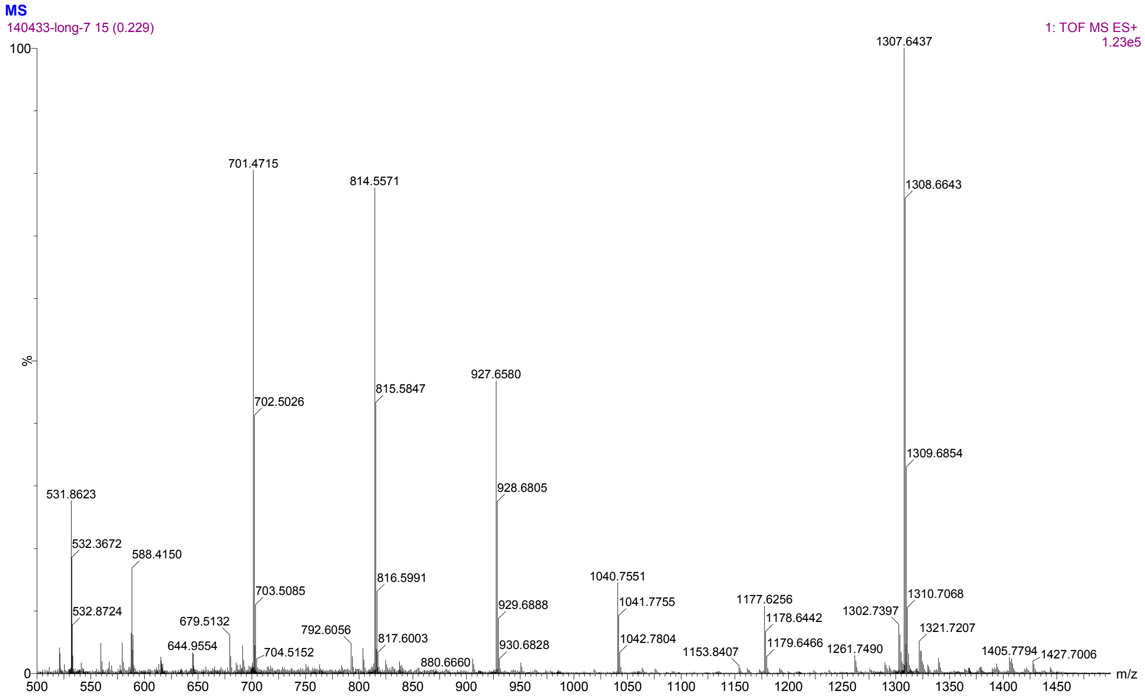

Figure S8. The HR-TOF-MS spectrum compounds **2**.

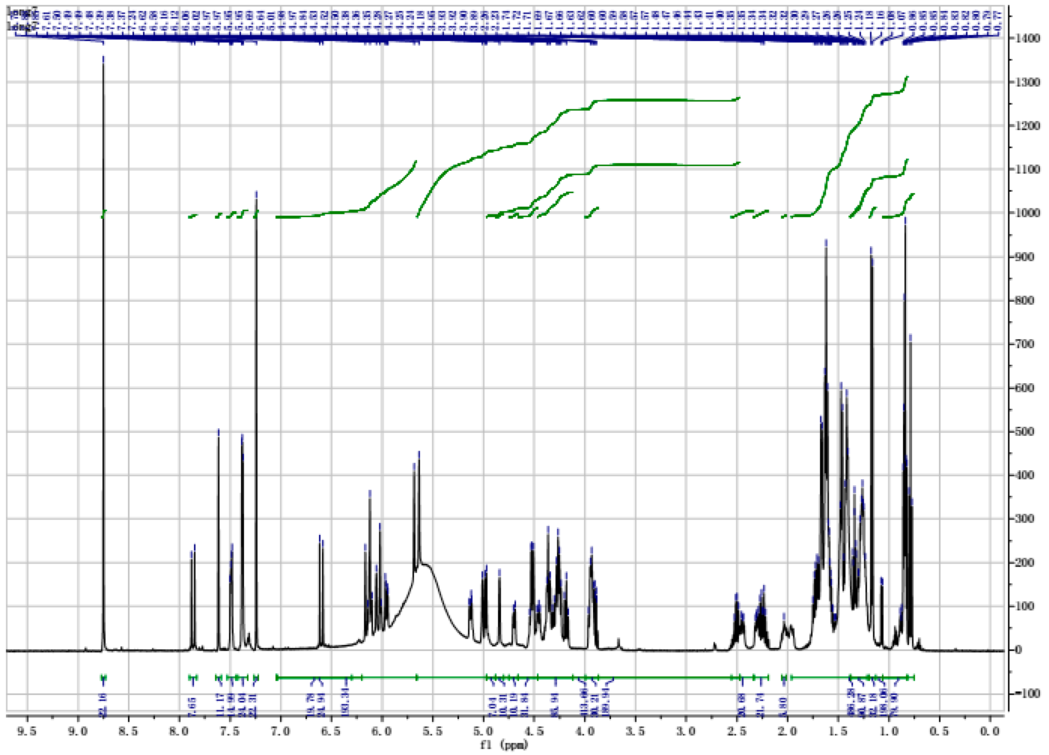

Figure S9. The ^{1}H-NMR spectrum compounds **2**.

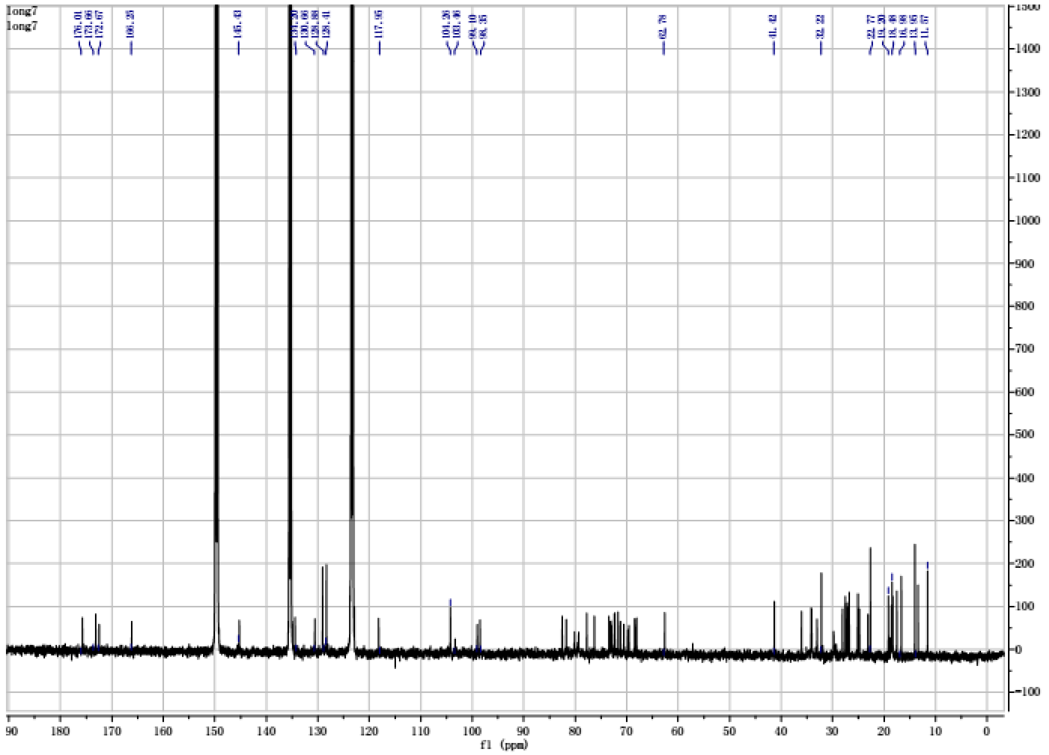

Figure S10. The ^{13}C-NMR spectrum compounds **2**.

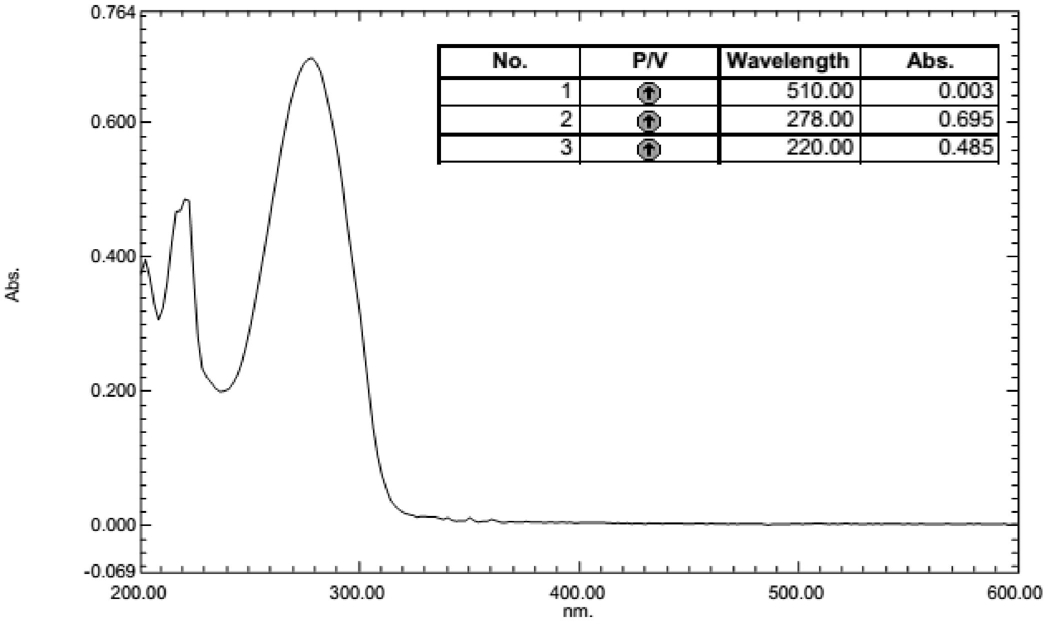

Figure S11. The UV spectrum compounds **3**.

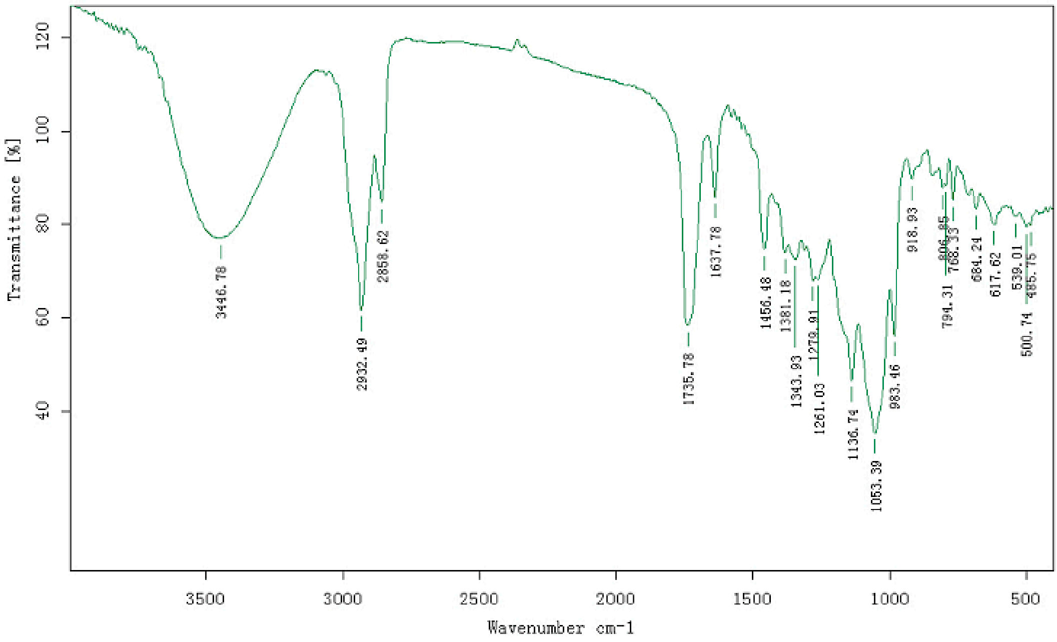

Figure S12. The IR spectrum compounds **3**.

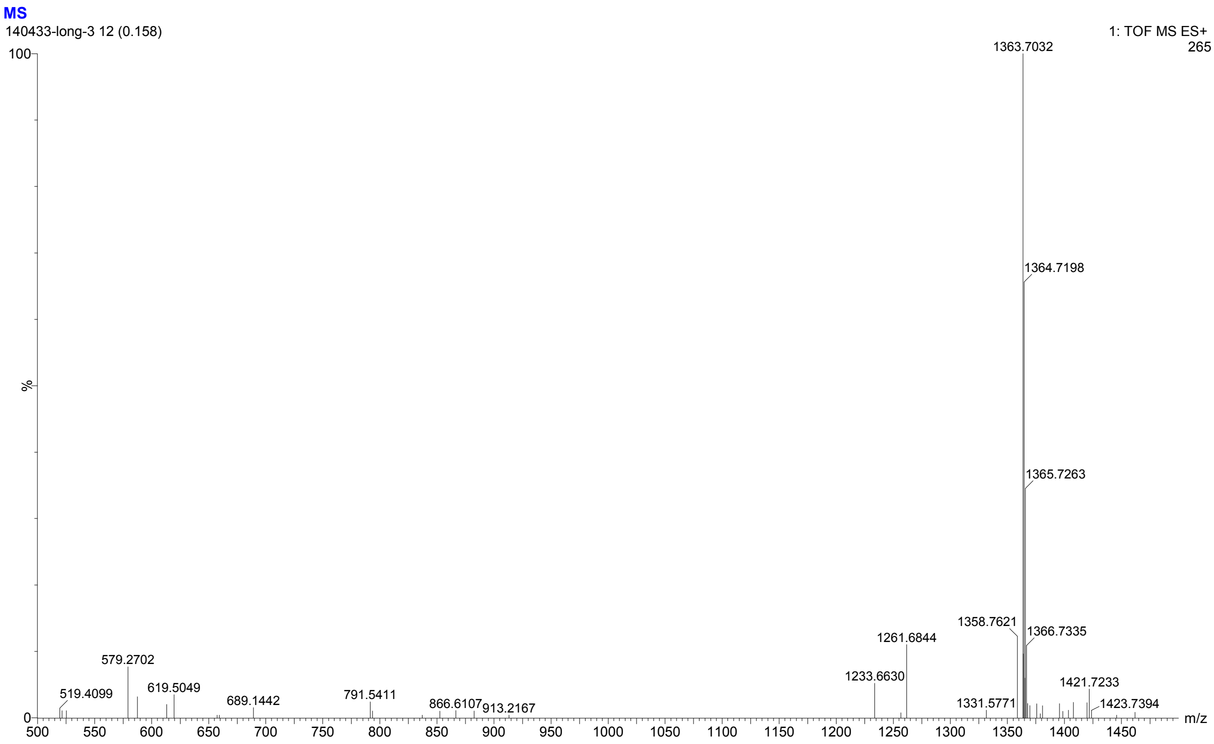

Figure S13. The HR-TOF-MS spectrum compounds **3**.

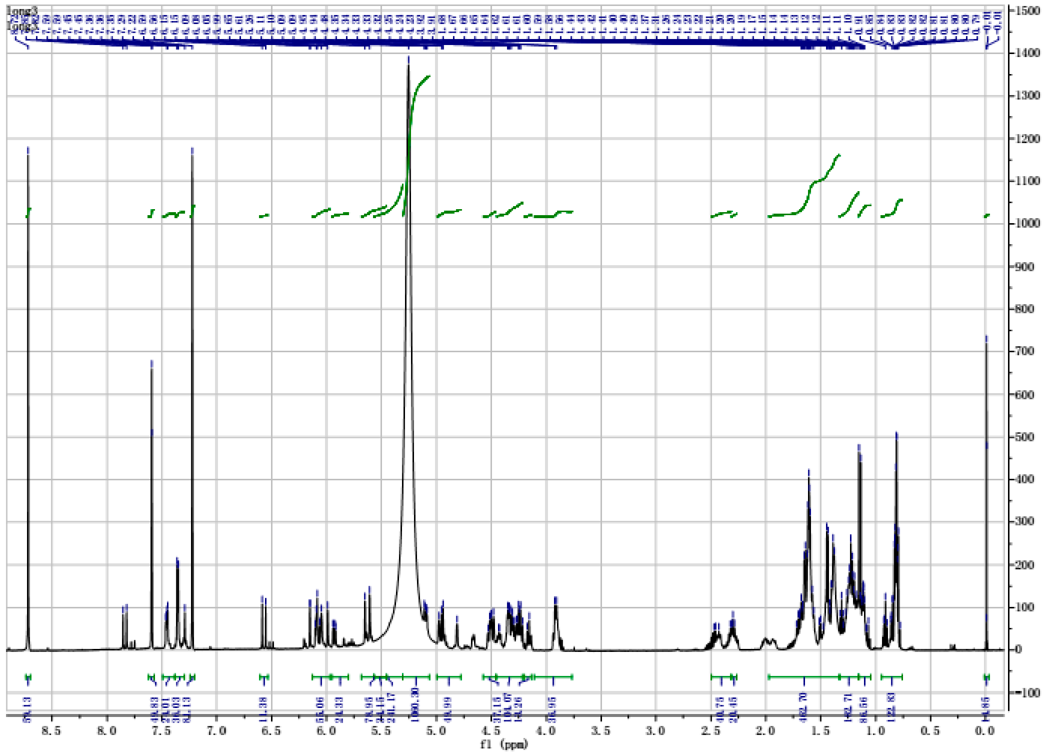

Figure S14. The ^{1}H-NMR spectrum compounds **3**.

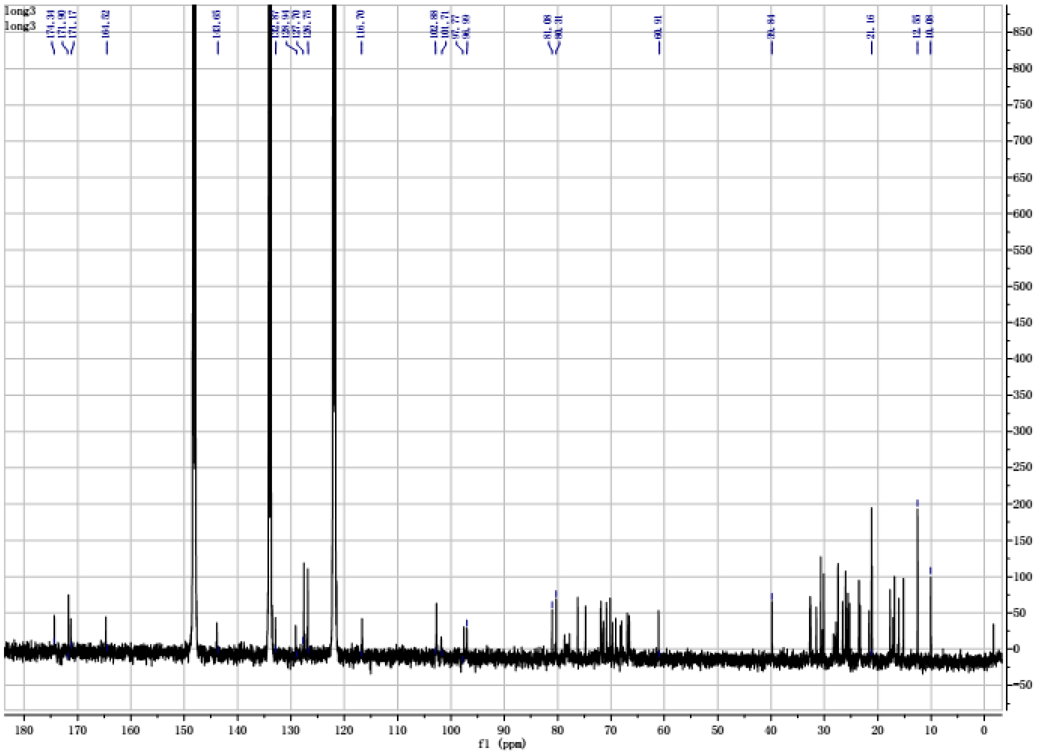

Figure S15. The ^{13}C-NMR spectrum compounds **3**.

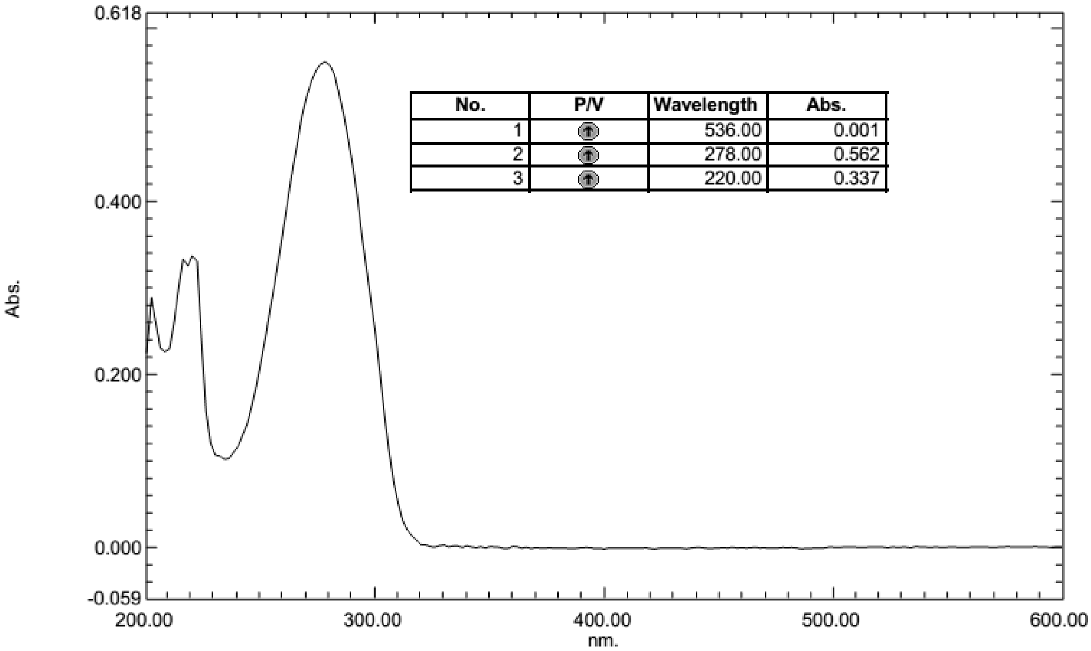

Figure S16. The UV spectrum compounds **4**.

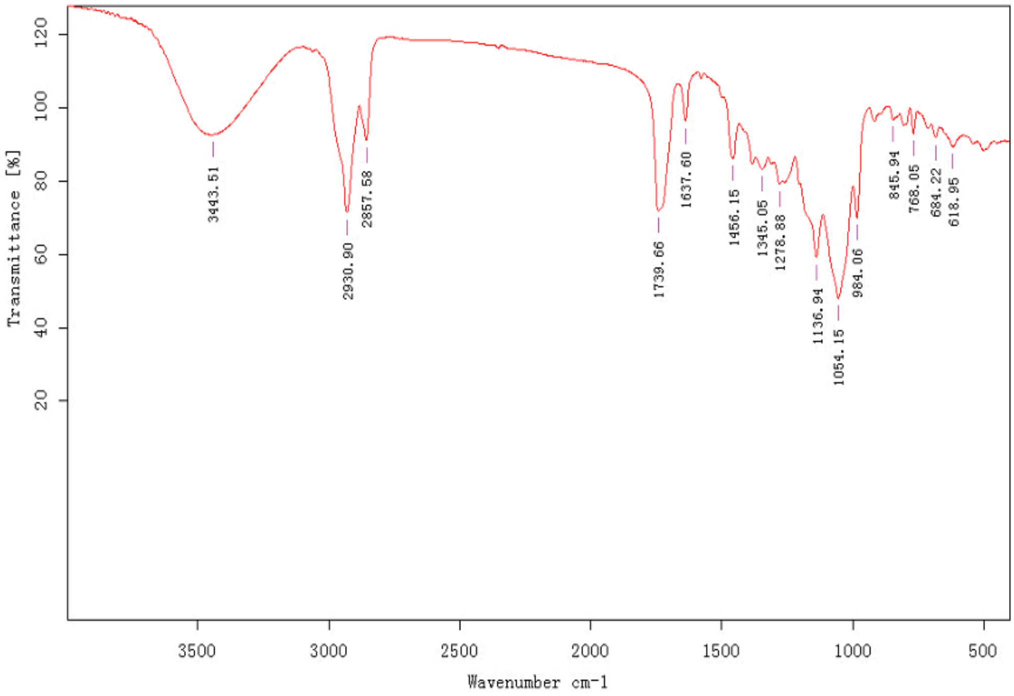

Figure S17. The IR spectrum compounds **4**.

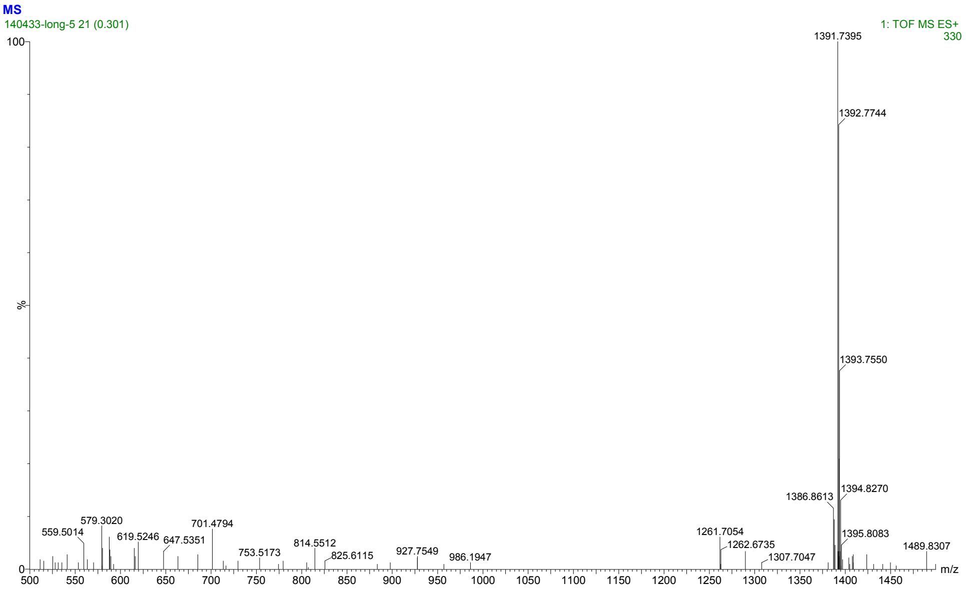

Figure S18. The HR-TOF-MS spectrum compounds **4**.

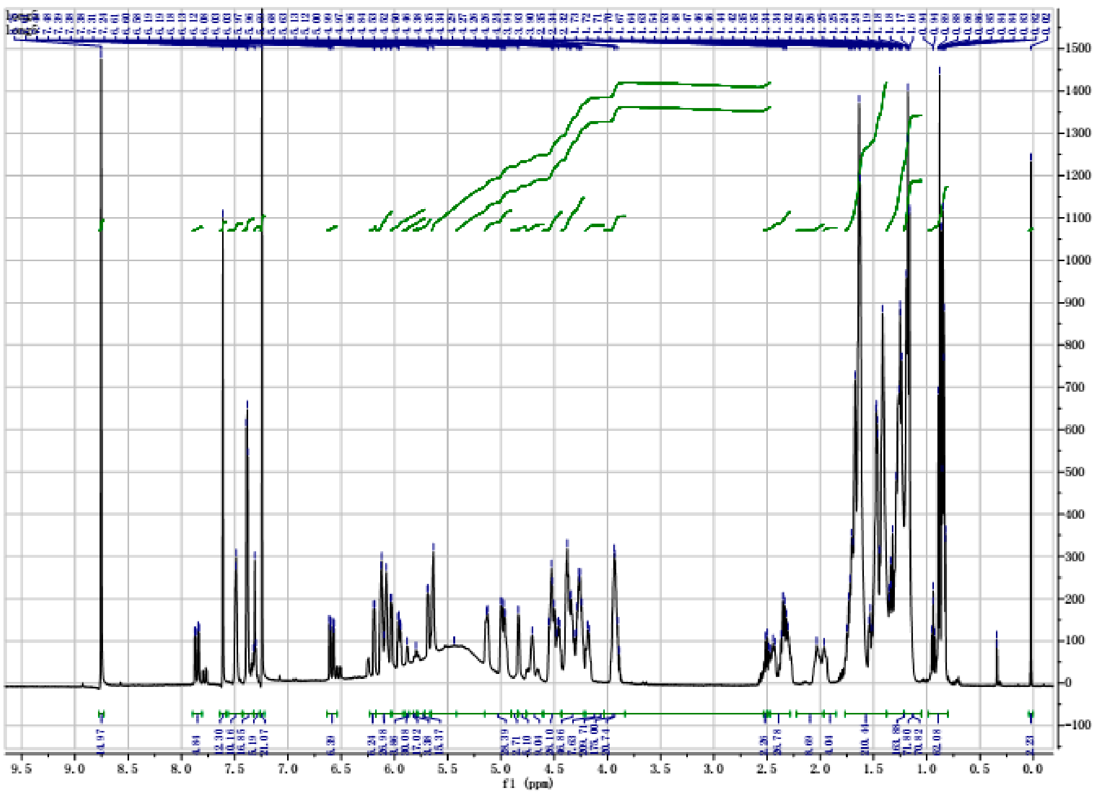

Figure S19. The ^{1}H-NMR spectrum compounds **4**.

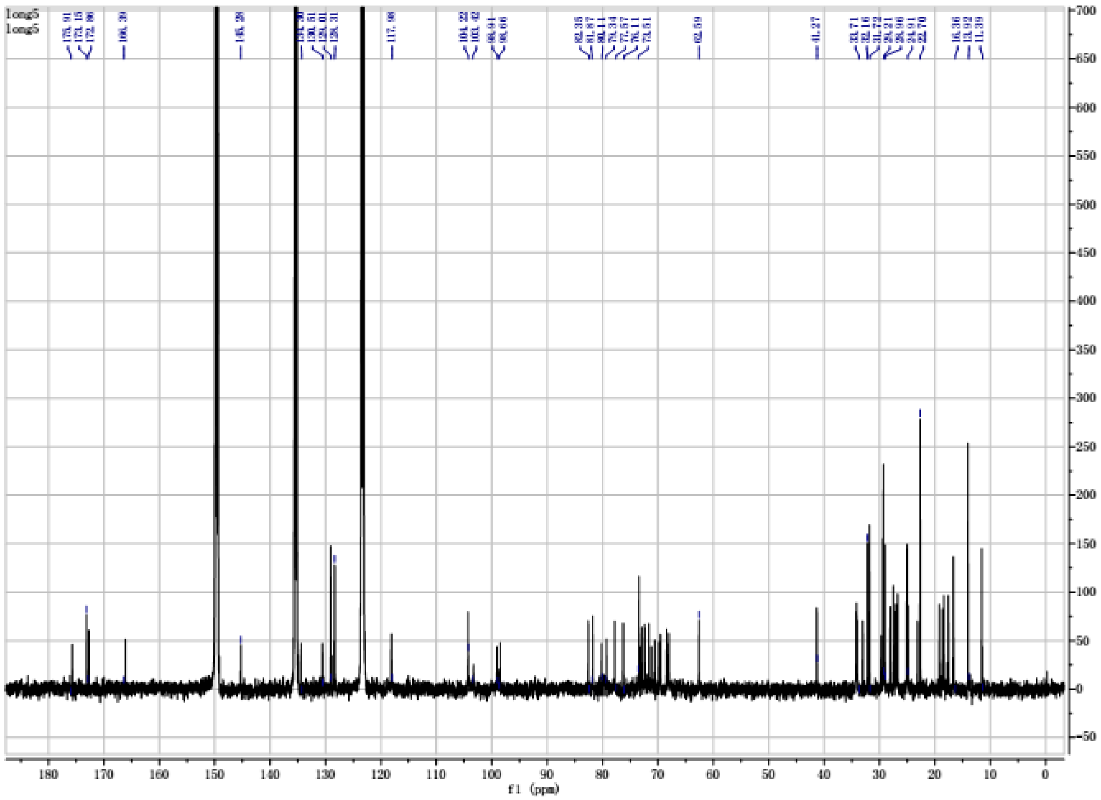

Figure S20. The ^{13}C-NMR spectrum compounds **4**.
